# Supplementary material for: Synthesis of high-entropy hydride from the cantor alloy (fcc–CoCrFeNiMn) at extreme conditions
Source: Nat Commun. 2026 Mar 17;17:2622. doi: 10.1038/s41467-026-70483-3 (PMC13002872; doi:10.1038/s41467-026-70483-3)
Supplement: Supplementary file 1 — Supplementary Information [file 41467_2026_70483_MOESM1_ESM.pdf]

SUPPLEMENTARY INFORMATION

Synthesis of High-Entropy Hydride from the Cantor Alloy (*fcc*–CoCrFeNiMn) at Extreme Conditions

Konstantin Glazyrin,[*1] Kristina Spektor,[*1] Maxim Bykov,[2]

Paulo H. B. Carvalho,[3] Weiwei Dong,[1‡] Fritz Körmann,[4,5,6]

Asami Sano-Furukawa,[7] Takanori Hattori,[7] Doreen C. Beyer,[8]

Martin Sahlberg,[3] Yuji Ikeda,[4] Ji Hun Yu,[9] Sangsun Yang,[9] Jai-Sung Lee,[10]

Shrikant Bhat,[1] Michael Hanfland,[11] Blazej Grabowski,[4]

Sergiy Divinski,[12] Kirill V. Yusenko[*13]

[1] Deutsches Elektronen-Synchrotron DESY, Notkestr. 85, Hamburg, 22607, Germany

[2] Institute for Inorganic and Analytical Chemistry, Goethe University Frankfurt, Max-von-Laue-str. 7, Frankfurt am Main, 60438, Germany

[3] Department of Chemistry - Ångström Laboratory, Uppsala University, Box 523, Uppsala, 75120, Sweden

[4] Institute for Materials Science, University of Stuttgart, Pfaffenwaldring 55, Stuttgart, 70569, Germany

[5] Department for Computational Materials Design, Max Planck Institute for Sustainable Materials GmbH, Max-Planck-Str.1, Düsseldorf, 40237 Germany

[6] Interdisciplinary Centre for Advanced Materials Simulation (ICAMS), Ruhr-Universität Bochum, Universitätsstr. 150, Bochum, 44801, Germany

[7] J-PARC Center, Japan Atomic Energy Agency, 2-4 Shirakata, Tokai-mura, Naka-gun, Ibaraki, 319-1195, Japan

[8] Leipzig University, Faculty of Chemistry, Institute of Inorganic Chemistry and Crystallography, Johannisallee 29, Leipzig, 04103, Germany

[9] Powder Materials Division, Korea Institute of Materials Science, Changwon, 51508, South Korea

[10] Department of Materials Science and Chemical Engineering, Hanyang University ERICA, 55 Hanyangdaehak-ro, Ansan, 15588, South Korea

[11] ESRF – The European Synchrotron, 71 Av. des Martyrs, Grenoble, 38000, France

[12] Institute of Materials Physics, University of Münster, Münster, 48149, Germany

[13] Institute of Geology, Mineralogy and Geophysics, Faculty of Geosciences, Ruhr-University Bochum, Universitätsstrasse 150, Bochum, 44801, Germany

*Contact emails: konstantin.glazyrin@desy.de, kristina.spektor@desy.de, kirill.yusenko@ruhr-uni-bochum.de

‡Current address: Beijing Synchrotron Radiation Facility (BSRF), Institute of High Energy Physics, Chinese Academy of Sciences, Beijing 100049, China

This document contains supplementary information to our experiments with diamond anvil cells (DACs), multi-anvil large volume press (LVP) and DFT calculations.

1. DAC experiments

In Figure S we show selected 2D patterns from our experiment DAC02 facilitating resistive heating setup. The patterns demonstrate initial phase purity and stability of *fcc*-Cantor high entropy alloy (HEA; *e.g.* Figure Sa). The patterns observed at higher pressures can be clearly indexed, as shown in the figure. However, due to increased diffuse scattering and a reduction in signal quality, particularly evident in the broadening of peaks and their overlap at high pressures, as well as the formation of a hydride phase, it was not possible to reliably determine the volumes of the *hcp* phases corresponding to the alloy and its *hcp* hydride. Nevertheless, indexing can be performed using the information provided in [1], including information on material compressibility and *c/a* unit lattice parameter ratio. Our data suggest that *c/a* ratio for the *hcp* hydride phase is similar to *hcp* of the precursor HEA implying homogeneous unit lattice expansion as a result of hydrogen absorption.

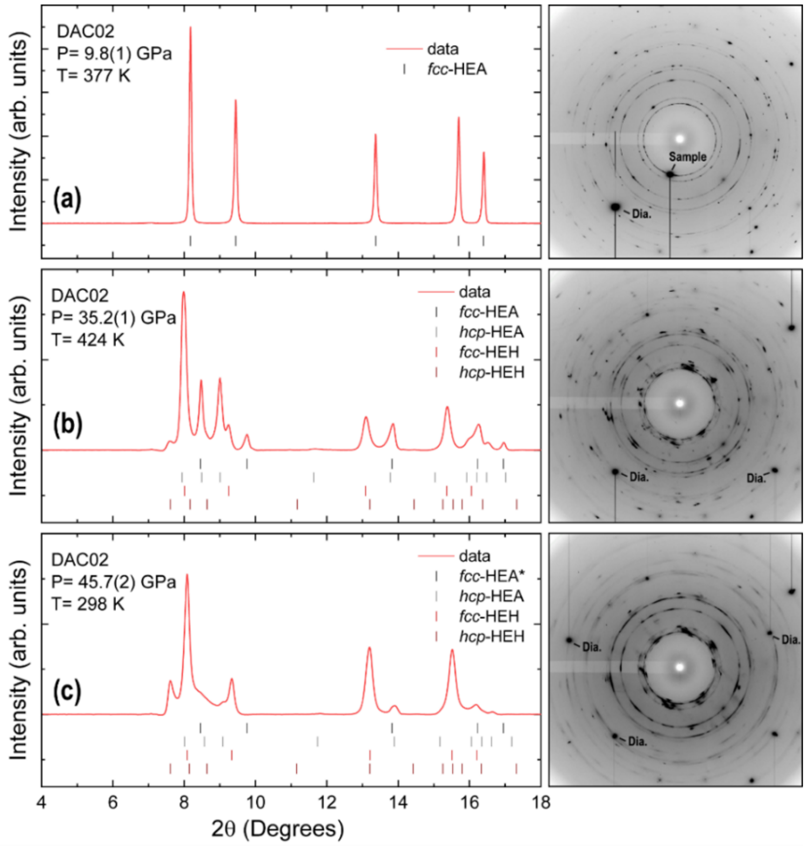

Figure S1 Selected diffraction patterns from diamond anvil cell experiment DAC02. Within the panels, the corresponding pressure–temperature (P–T) conditions for the displayed diffractograms are indicated, along with the indexing of the high-entropy alloy (HEA) and high-entropy hydride (HEH) polymorphs. The experiment was conducted at beamline P02.2 of PETRA III at DESY (Hamburg, Germany), using a wavelength of $\lambda = 0.2907$ Å. In panel (c), a star symbol (*) indicates a potentially negligible presence of the *fcc*-HEA phase at 45.7(2) GPa. To simplify representation, background of 1D patterns was subtracted by means of DIOPTAS software package [2] The right-hand panels show 2D diffractograms collected with a PerkinElmer XRD1621 detector, corresponding to the 1D patterns displayed in the left-hand panels. Detector artifacts and strong reflections from the sample or diamonds, as selectively indicated in the 2D patterns, were masked during signal integration. Red lines represent 1D patterns of the raw experimental data

During the DAC02 experiment we could heat up to 424 K. The temporal profiles regarding pressure and temperature variations are shown in Figure S.

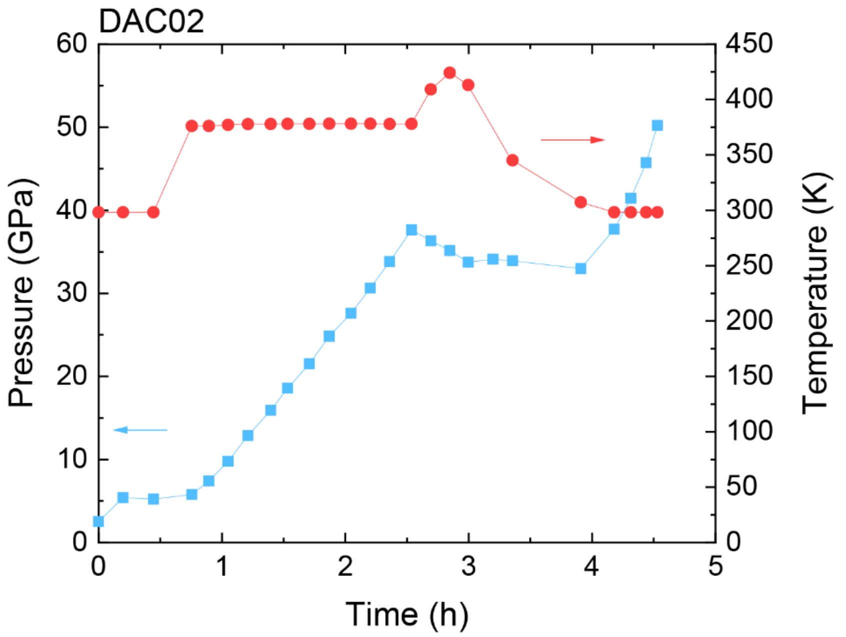

Figure S2 Temporal profiles of pressures and temperatures attributed to the experiment DAC02. Note that during the heating from 376-378 to 424 K, pressure of the sample chamber was slightly dropping.

The pure transition elements and their alloys can form hydrides. The formation of the associated hydrides is controlled by thermodynamic conditions and kinetics. Here we show a typical example of Re gasket loaded initially with a high entropy alloy and hydrogen in a DAC. The loading was compressed to 5 GPa at ambient temperature, it was then heated to 200 °C using a whole cell resistive heating setup of P02.2, PETRA–III, DESY and further compressed to 18 GPa. During this, process hydrogen dissociated at the boundary with Re and then diffused into the bulk of the metal. Similar is the process of hydrogenation of the Cantor alloy.

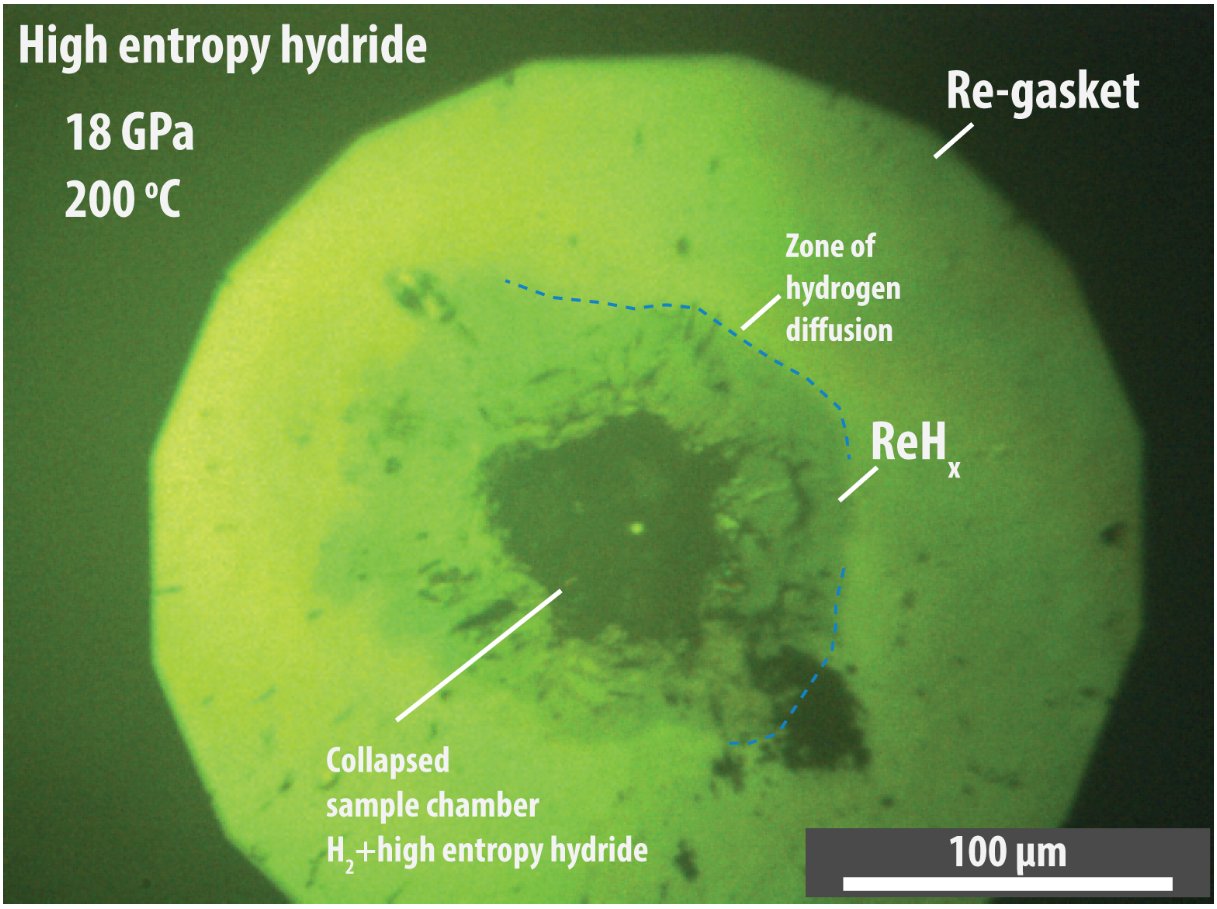

Figure S3 Microphotograph of a DAC loading with high-entropy hydride at 18 GPa and 200 °C showing Re gasket hydrogenation at elevated temperatures. Significant diffusion of hydrogen through the gasket material leads to a collapse of the indicated sample chamber. Formation of ReH_x within the gasket material can be easily verified using X-ray diffraction.

2. LVP experiments

Here we present supplementary information attributed to the multi-anvil experiment LVP01 conducted at P61B, PETRA III, DESY, Hamburg. In Figure S4 we show time profiles of temperature and pressure conditions of our experiments, while in Figure S5 we show examples of energy dispersive diffraction patterns collected at the same beamline.

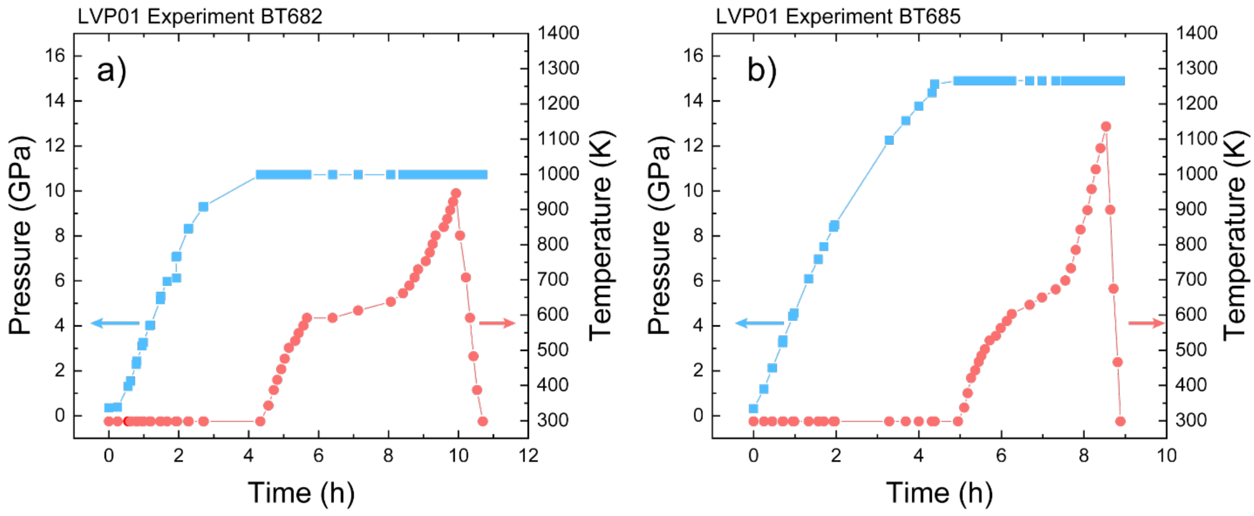

Figure S4 Pressure and temperature time profiles of multi-anvil experiments (LVP01). Panel a) shows data from the LVP01 experiment at 10.7 GPa and ~950 K, corresponding to the highest pressure and temperature, respectively. Panel b) presents data from another LVP01 experiment conducted at 14.9 GPa and ~1140 K, again representing the highest pressure and temperature reached. Cyan and rose colors are used to indicate pressure and temperature values, respectively. Colored lines are provided as visual guides.

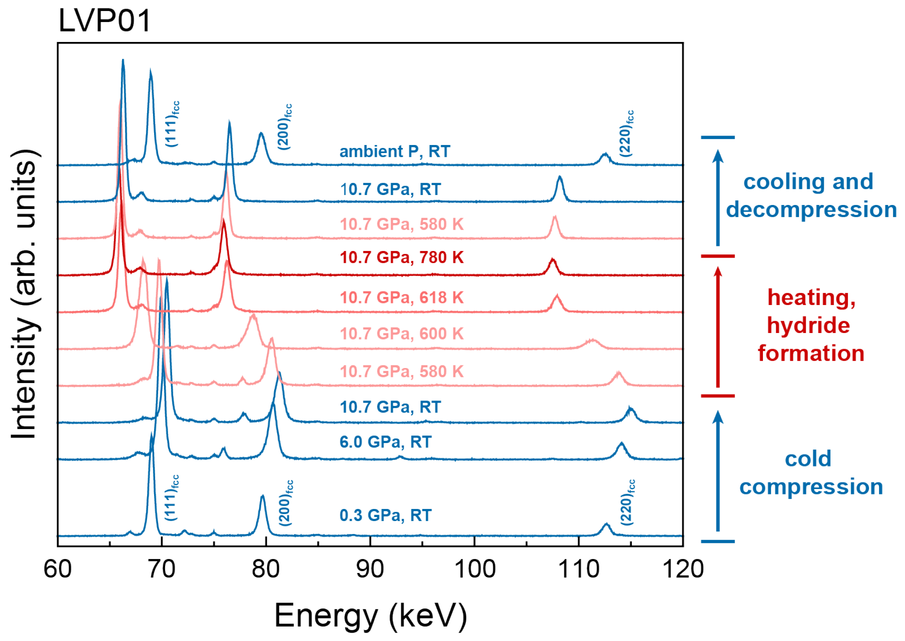

Figure S5 Selected patterns collected at energy dispersive diffraction beamline P61B (with detector diffracted angle 2θ=4.9621°). The patterns indicated in the figure correspond to pressure points shown in Figure S4. The patterns clearly reflect the process of hydrogenation, with the material volume increasing under pressure due to the reaction with hydrogen formed from the decomposition of NH_3BH_3. Peaks corresponding to the *fcc* Cantor alloy are indicated for the patterns attributed to 0.3 GPa and ambient conditions. Small additional peaks in the latter diffractogram, aside from the alloy signal, can be attributed to undesirable signal contribution. For example, we observed minor X-ray fluorescence signal which could be attributed to the contribution from the experimental hutch shielding (*e.g.* lead with $K_{\alpha 2}^{Pb} = 72.80\ keV$; $K_{\alpha 1}^{Pb} = 74.97\ keV$; $K_{\beta 1}^{Pb} = 84.94\ keV$). The blue, red and pink lines represent 1D patterns of the raw experimental data.

3. DFT calculations

In addition to the results discussed in the main text of the manuscript, in Figure S6 we show the local moment distribution obtained from five special quasirandom structures (SQSs) for the *fcc*, *hcp* phases as well as their octahedral hydrides calculated for ambient pressure conditions.

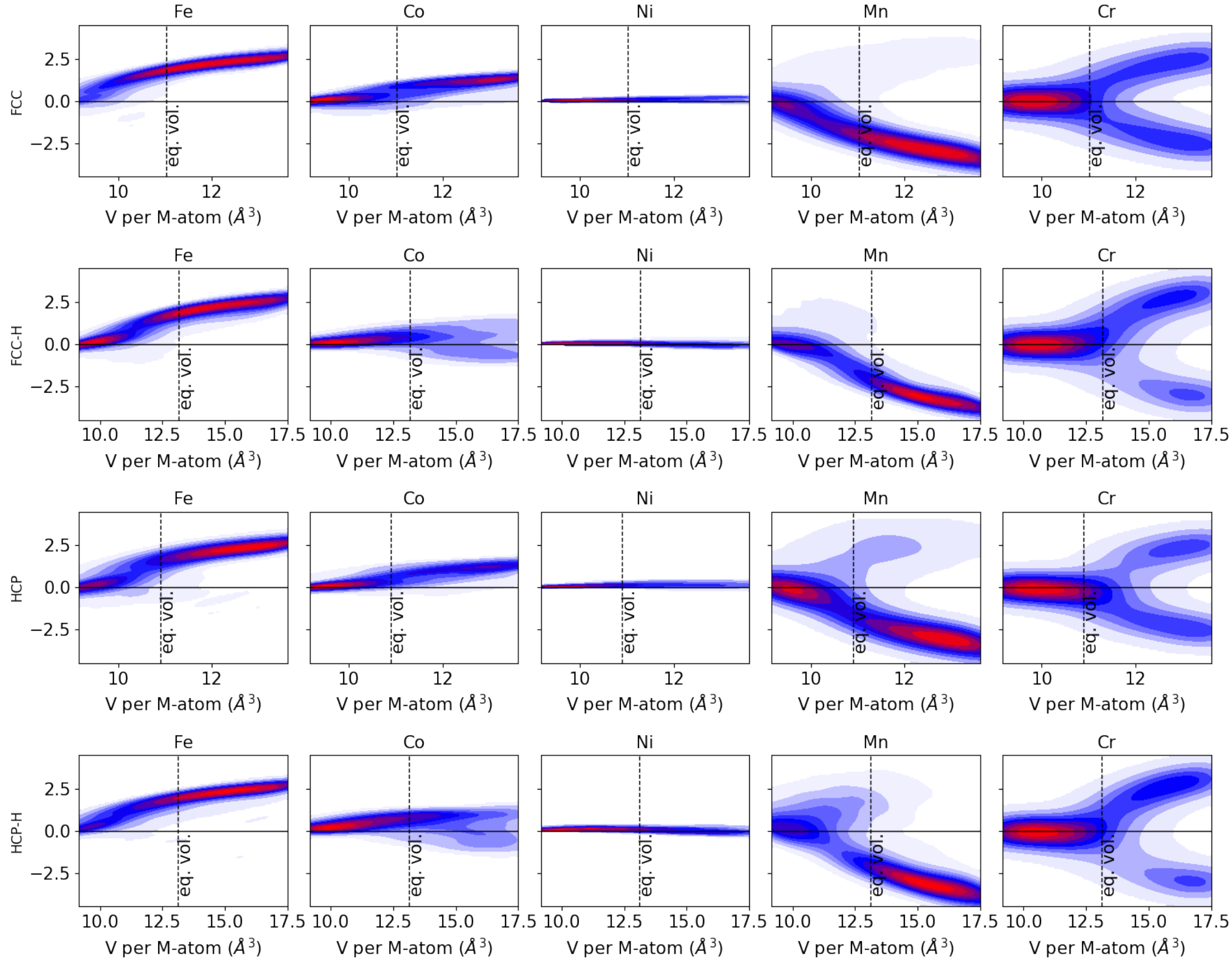

Figure S6 Results of local magnetic moment distribution calculation. The calculations were conducted for the conditions corresponding to 0 GPa and 0 K. The *x*-axis and *y*-axis values of individual panels correspond to volume per metal atom in units of Å^3 and magnetic moment of the atom in units of μ_B (Bohr magneton), respectively. The local magnetic moment distribution after the self-consistent electronic minimization in the *fcc* and *hcp* Cantor alloy (denoted as FCC and HCP), as well as their hydride phases with octahedral sites filled by hydrogen (denoted as FCC–H and HCP–H), are shown. The dashed line corresponds to the equilibrium volume values for the *P*–*T* conditions of the calculation.

4. Relative stability of octahedral and tetrahedral occupancies

To clarify the hydrogen site preference at high pressures, additional DFT calculations were performed. Two distinct "half-filled" (checkerboard-like) tetrahedral hydride configurations (composition M:H=1:1) were examined and compared with the octahedral configuration. Both tetrahedral configurations yielded essentially the same result, confirming the preference of dissociated hydrogen to octahedral sites. The results for one of them are summarized in Figure S7. The calculations indicate that the octahedral site remains energetically preferred at all pressures examined. The tetrahedral configurations exhibit substantially larger equilibrium volumes and higher total energies, which are inconsistent with experimental observations. This further confirms that the hydride adopts an octahedral occupancy.

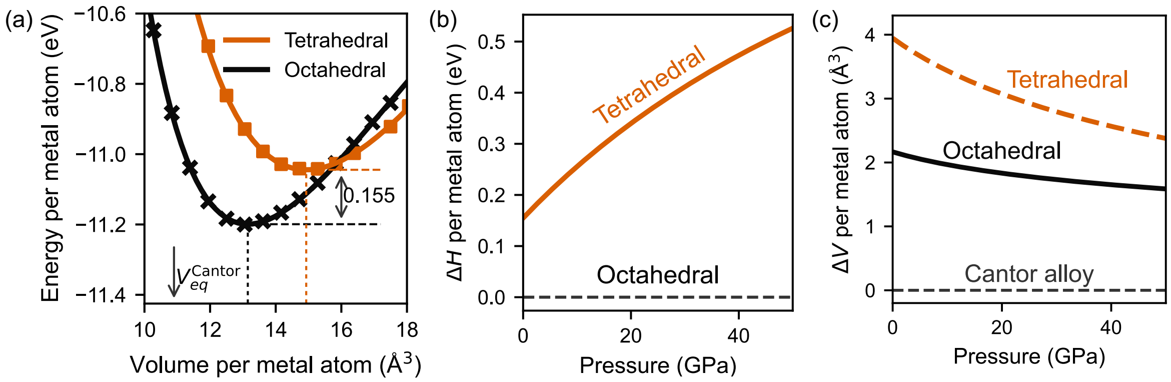

Figure S7 (a) total energy per metal atom versus volume per metal atom for octahedral and tetrahedral configurations in hydrogenated Cantor alloy, (b) the enthalpy difference between octahedral and tetrahedral occupancies under pressure, and (c) volume change as a function of pressure.

References

1. Tracy, C. L. *et al.* High pressure synthesis of a hexagonal close-packed phase of the high-entropy alloy CrMnFeCoNi. *Nature Communications, 2017,* **8**, 1–6

2. Prescher, C. & Prakapenka, V. B. DIOPTAS: a program for reduction of two-dimensional X-ray diffraction data and data exploration, 2015, **35**, 223–230, *http://dx.doi.org/10.1080/08957959.2015.1059835*
